# Chiral and Molecular Recognition through Protonation between Aromatic Amino Acids and Tripeptides Probed by Collision-Activated Dissociation in the Gas Phase

**DOI:** 10.3390/molecules23010162

**Published:** 2018-01-13

**Authors:** Akimasa Fujihara, Hikaru Inoue, Masanobu Sogi, Michiko Tajiri, Yoshinao Wada

**Affiliations:** 1 Department of Chemistry, Osaka Prefecture University, Osaka 599-8531, Japan; h_inoue@c.s.osakafu-u.ac.jp (H.I.), sv274214350@yahoo.co.jp (M.S.); 2 Department of Molecular Medicine, Osaka Women’s and Children’s Hospital, Osaka 594-1101, Japan; plaza-tajiri@mch.pref.osaka.jp (M.T.), waday@mch.pref.osaka.jp (Y.W.)

**Keywords:** chemical evolution, molecular clouds, enantiomeric excess, homochirality

## Abstract

Chiral and molecular recognition through protonation was investigated through the collision-activated dissociation (CAD) of protonated noncovalent complexes of aromatic amino acid enantiomers with l-alanine- and l-serine-containing tripeptides using a linear ion trap mass spectrometer. In the case of l-alanine-tripeptide (AAA), NH_3_ loss was observed in the CAD of heterochiral H^+^(d-Trp)AAA, while H_2_O loss was the main dissociation pathways for l-Trp, d-Phe, and l-Phe. The protonation site of heterochiral H^+^(d-Trp)AAA was the amino group of d-Trp, and the NH_3_ loss occurred from H^+^(d-Trp). The H_2_O loss indicated that the proton was attached to the l-alanine tripeptide in the noncovalent complexes. With the substitution of a central residue of l-alanine tripeptide to l-Ser, ASA recognized l-Phe by protonation to the amino group of l-Phe in homochiral H^+^(l-Phe)ASA. For the protonated noncovalent complexes of His enantiomers with tripeptides (AAA, SAA, ASA, and AAS), protonated His was observed in the spectra, except for those of heterochiral H^+^(d-His)SAA and H^+^(d-His)AAS, indicating that d-His did not accept protons from the SAA and AAS in the noncovalent complexes. The amino-acid sequences of the tripeptides required for the recognition of aromatic amino acids were determined by analyses of the CAD spectra.

## 1. Introduction

Biomolecules have the ability to recognize chiral molecules. Enantiomeric separation is crucial in chemistry, because one enantiomer of a chiral drug may be toxic to biological systems, while another is medically effective. Analytical techniques such as high-performance liquid chromatography, capillary electrophoresis, gas chromatography, nuclear magnetic resonance (NMR) spectroscopy, X-ray crystallography, and circular dichroism are used to distinguish between the enantiomers of chiral molecules [1,2,3,4]. Mass spectrometry-based techniques for chiral recognition using chiral host molecules have been developed over the past two decades, and are highly sensitive and suitable for analyzing mixtures [5,6,7,8,9]. Recently, the chiral differentiation of amino acids by the ion mobility mass spectrometry [10,11,12] and NMR spectroscopy of gas-phase ions using magnetic resonance acceleration [13,14] has been reported. 

Chiral recognition in biological systems is attributed to homochirality in biomolecules consisting of l-amino acids in proteins and d-(deoxy) ribose in nucleic acids. The chiral recognition phenomena and origin of homochirality in biomolecules represent one of the most interesting fields of scientific research regarding the origin of life on Earth [15,16,17]. Miller synthesized amino acids from simple compounds under possible primitive Earth conditions [18]. Amino acids and peptides were formed by the ultraviolet or electron irradiation of interstellar molecules condensed on a cold surface, suggesting the abiotic formation of biological molecules under extraterrestrial conditions [19,20,21,22,23]. However, no enantiomeric excess has been observed.

The excess l-amino acids found in the Murchison meteorite [24,25,26] and the chiral molecule propylene oxide observed in a star-forming region [27] suggest an extraterrestrial origin for enantiomeric excess. The circularly polarized light found in star-formation regions suggests the possibility of enantioselective photodissociation forming enantiomeric excess in interstellar space [28]. A hypothesis for the extraterrestrial origin of biomolecules has been proposed based of these studies. In this hypothesis, racemic amino acids formed in interstellar molecular clouds, followed by the enantioselective photodissociation of d-enantiomers with circularly polarized light [15,16,17].

We investigated the structure and reactivity of mass-selected and temperature-controlled gas-phase noncovalent complexes containing biological molecules, as a model of interstellar molecular clouds. The enantiomer-selective photodissociation of cold gas-phase protonated d-tryptophan H^+^(d-Trp) on a chiral crown ether was suppressed at temperatures greater than 170 K, and no reactivity difference was observed between the d- and l-enantiomers at 300 K [29,30]. The temperatures of the gas-phase noncovalent complexes corresponded to those of interstellar and atmospheric molecular clouds. When three l-serine are present in a protonated noncovalent complex, the photodissociation of Trp in the noncovalent complex is enantiomer-selective [31]. The photo-excitation of cold gas-phase noncovalent complexes between protonated Trp and disaccharides showed that photo-induced *C*-glycosylation could occur in interstellar molecular clouds, with its enantiomeric selectivity depending on the structure of the disaccharide [32]. Enantiomer-selective photodissociation in the gas phase was used for the quantitative chiral analyses of biological molecules in solution [33,34].

Collisional activation is a universal process in the interstellar environment, and an enantiomer-selective reaction is important to formulate a hypothesis for the origin of homochirality in biomolecules. Therefore, we previously investigated the collision-activated dissociation (CAD) of gas-phase protonated noncovalent complexes between Trp enantiomers and l-alanine peptides as a function of the peptide size. The CAD spectra indicated that chiral recognition through protonation by l-alanine tripeptide induces the enantiomer-selective CAD of Trp [35]. Photodissociation experiments also showed the chiral recognition ability of l-alanine tripeptide for Trp [36].

In this study, we examined chiral and molecular recognition based on protonation between aromatic amino acids and l-alanine- and l-serine-containing tripeptides through the CAD of gas-phase protonated noncovalent complexes between aromatic amino acids and peptides. Trp, Phe, Tyr, and His contain indole, benzene, phenol, and imidazole side chains, respectively. Ser was formed by the ultraviolet irradiation of interstellar ice analogs [19,20], and showed enantiomer-selectivity in the gas phase [31,37,38]. Based on the results, we determined the amino-acid sequences of tripeptides needed for the recognition of aromatic amino acids.

## 2. Results and Discussion

### 2.1. Chiral Recognition Ability of l-Alanine Peptides

In the mass spectra of the gas-phase ions generated using nanoelectrospray ionization, single-charged ions such as protonated amino acids, protonated tripeptides, and protonated 1:1 noncovalent complexes between amino acids and tripeptides were observed. Stable magic number clusters as observed in case of Ser [31,38] and a difference between enantiomers were not observed in the mass spectra.

**Figure 1:**
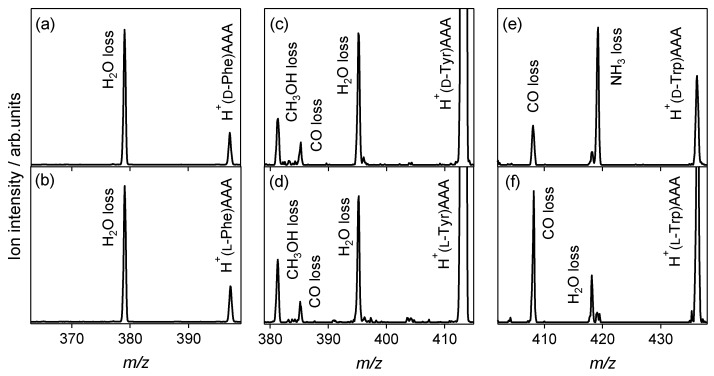
Collision-activated dissociation spectra of protonated noncovalent complexes of aromatic amino acids with l-alanine tripeptide: (**a**) H^+^(d-Phe)AAA, (**b**) H^+^(l-Phe)AAA, (**c**) H^+^(d-Tyr)AAA, (**d**) H^+^(l-Tyr)AAA, (**e**) H^+^(d-Trp)AAA, and (**f**) H^+^(l-Trp)AAA.

Figure 1 shows the CAD spectra of the protonated noncovalent complexes of aromatic amino acids with l-alanine tripeptide. The spectra of heterochiral H^+^(d-Trp)AAA and homochiral H^+^(l-Trp)AAA are also shown for comparison, which are cited from our previous paper [35]. For the Phe and Tyr shown in Figure 1a–d, the H_2_O loss from the noncovalent complex is the main dissociation pathway, and no reactivity difference between the enantiomers is observed in the spectra. For the heterochiral H^+^(d-Trp)AAA shown in Figure 1e, the NH_3_ loss is the main dissociation pathway, whereas the H_2_O-elimination product is observed in the spectrum of homochiral H^+^(l-Trp)AAA, as with Phe and Tyr. The loss of either NH_3_ or H_2_O in the CAD is attributed to the protonation site in the noncovalent complex [35]. The protonation site of heterochiral H^+^(d-Trp)AAA is the amino group of d-Trp, and the NH_3_ loss occurs from H^+^(d-Trp). The NH_3_ loss from the protonated aromatic amino acids is the primary dissociation pathway in CAD [39]. The H_2_O losses observed in all the spectra except that for d-Trp indicate that the proton is attached to the l-alanine tripeptide in each noncovalent complex. H_2_O loss is the main pathway for the CAD of protonated polyalanines, where the NH_3_-elimination product is not observed [40]. The dehydration occurs at the carbonyl group or amino bond of the protonated polyalanines.

CAD experiments using the l-alanine di-, tetra-, and hexa-peptides were also performed for Phe, Tyr, and His. The H_2_O-elimination product is observed in all the spectra of the protonated noncovalent complexes of the aromatic amino acid enantiomers with l-alanine peptides, as in the case for Trp reported previously [35]. The NH_3_-elimination product is not observed, and no difference between enantiomers is observed in the spectra. Only the l-alanine tripeptide recognizes d-Trp from the aromatic amino acids through protonation to the amino group of d-Trp. An enantiomeric excess of l-Trp is formed by the NH_3_ loss of H^+^(d-Trp) in the noncovalent complex, enantiomer-selective CAD, as discussed in our previous paper [35]. In addition to the NH_3_ and H_2_O losses, CH_3_OH and CO losses from the noncovalent complexes are observed for Tyr, and a CO loss is observed for Trp. The dissociations do not occur for Phe, where the H_2_O loss is the dissociation pathway. The reactivity in the noncovalent complexes depends on the species of aromatic amino acids, but does not indicate enantiomer-selectivity. The NH_3_ and H_2_O losses in CAD reflect chiral and molecular recognition through protonation in gas-phase noncovalent complexes.

### 2.2. Chiral and Molecular Recognition of l-Serine-Containing Tripeptides

#### 2.2.1. Tryptophan and Phenylalanine

**Figure 2:**
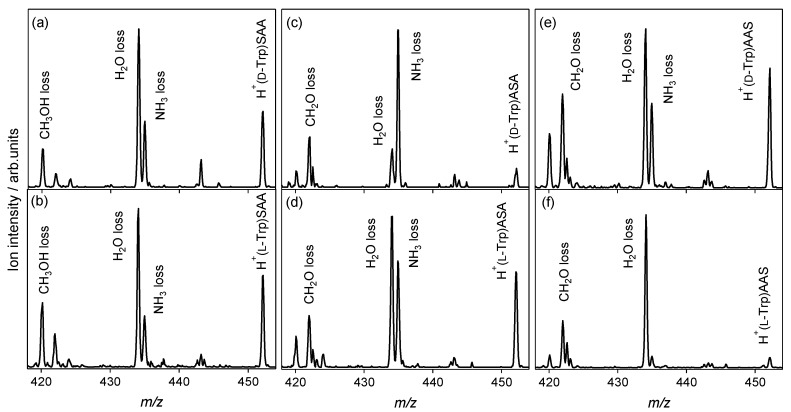
Collision-activated dissociation spectra of protonated noncovalent complexes of tryptophan with l-serine-containing tripeptides: (**a**) H^+^(d-Trp)SAA, (**b**) H^+^(l-Trp)SAA, (**c**) H^+^(d-Trp)ASA, (**d**) H^+^(l-Trp)ASA, (**e**) H^+^(d-Trp)AAS, and (**f**) H^+^(l-Trp)AAS (*m*/*z* 452).

CAD experiments with protonated noncovalent complexes of aromatic amino acid enantiomers with l-serine-containing tripeptides were performed to investigate the relationships between the chiral recognition ability of tripeptides and their amino-acid sequences. Figure 2 shows the CAD spectra of protonated noncovalent complexes of Trp enantiomers with l-serine-containing tripeptides. SAA and AAS are the tripeptides, where the N- and C-terminal residues at the end of the tripeptides are l-Ser. In the CAD spectra of heterochiral H^+^(d-Trp)SAA and homochiral H^+^(l-Trp)SAA shown in Figure 2a,b, respectively, the NH_3_ and H_2_O losses are both observed, and similar spectra are obtained. The replacement of N-terminal l-Ala with l-Ser eliminates the chiral recognition ability of the tripeptide for Trp. For ASA and AAS, the NH_3_ and H_2_O losses are both observed in the spectra, and the enantiomers are distinguishable in the ratio of the ion intensity for the NH_3_ loss and H_2_O loss. However, the chiral recognition ability for Trp is decreased by the replacement of one residue of the l-alanine tripeptide with l-Ser. It is not possible to determine whether the isomers coexisted or proton sharing occurred between the Trp and l-serine-containing tripeptides in the noncovalent complexes simply from the CAD spectra. The chirality of Trp is highly recognized by the l-alanine tripeptide compared to the l-serine-containing tripeptides, as shown in Figure 1e,f.

**Figure 3:**
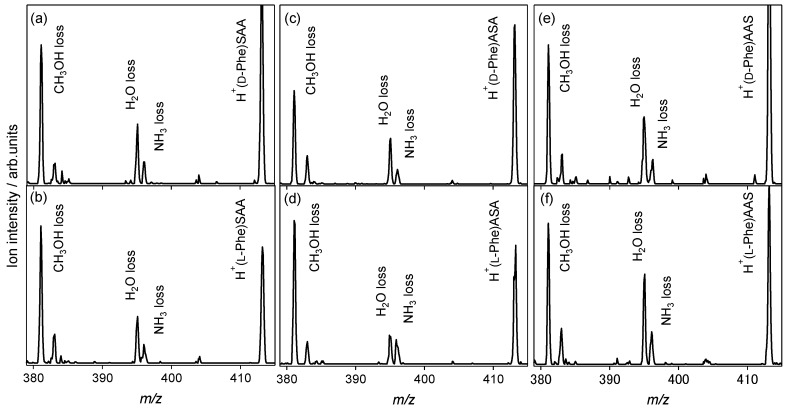
Collision-activated dissociation spectra of protonated noncovalent complexes of phenylalanine with l-serine-containing tripeptides: (**a**) H^+^(d-Phe)SAA, (**b**) H^+^(l-Phe)SAA, (**c**) H^+^(d-Phe)ASA, (**d**) H^+^(l-Phe)ASA, (**e**) H^+^(d-Phe)AAS, and (**f**) H^+^(l-Phe)AAS (*m*/*z* 413).

Figure 3 shows the CAD spectra of the protonated noncovalent complexes of the Phe enantiomers with l-serine-containing tripeptides. The NH_3_ loss is observed in addition to the H_2_O loss, whereas only the H_2_O-elimination product is observed in the case of the l-alanine tripeptide, as shown in Figure 1a,b. For the SAA and AAS, where the end of the l-alanine tripeptide is replaced by l-Ser, no difference between the enantiomers is observed in the spectra. However, the spectra of heterochiral H^+^(d-Phe)ASA and homochiral H^+^(l-Phe)ASA are distinguishable from the ratio of the ion intensity for the NH_3_ loss and H_2_O loss. The relative ion intensity of the NH_3_-elimination product to the H_2_O-elimination product of the homochiral H^+^(l-Phe)ASA, as shown in Figure 3d, is higher than that of the heterochiral H^+^(d-Phe)ASA, as shown in Figure 3c. This indicates that the proton affinity of l-Phe is higher than that of d-enantiomer when noncovalently complexed with ASA, because the NH_3_ loss is the dissociation pathway of the protonated aromatic amino acids. The ASA recognizes Phe and its chirality through protonation, whereas the AAA, SAA, and AAS do not have a chiral recognition ability for Phe.

#### 2.2.2. Tyrosine and Histidine

**Figure 4:**
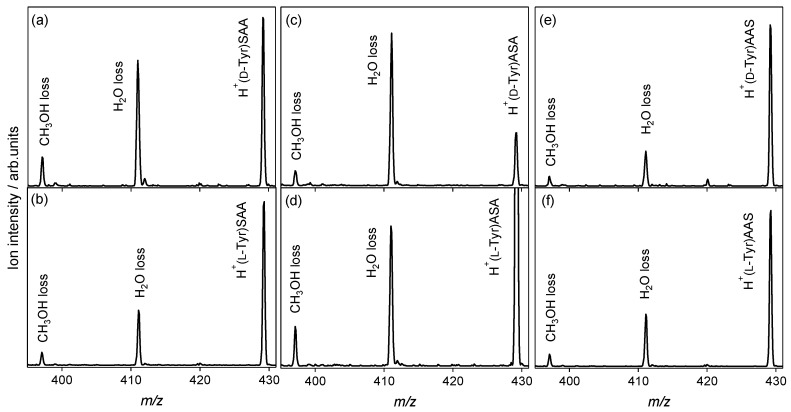
Collision-activated dissociation spectra of protonated noncovalent complexes of tyrosine with l-serine-containing tripeptides: (**a**) H^+^(d-Tyr)SAA, (**b**) H^+^(l-Tyr)SAA, (**c**) H^+^(d-Tyr)ASA, (**d**) H^+^(l-Tyr)ASA, (**e**) H^+^(d-Tyr)AAS, and (**f**) H^+^(l-Tyr)AAS (*m*/*z* 429).

In the CAD spectra of the protonated noncovalent complexes of the Tyr enantiomers with l-serine-containing tripeptides shown in Figure 4, the H_2_O loss from the noncovalent complexes is the main dissociation pathway, as in the case of the l-alanine tripeptide shown in Figure 1c,d. The NH_3_-elimination product is not observed, and no reactivity difference between the enantiomers is observed in the spectra. The l-serine-containing tripeptides and l-alanine peptides used in this study could not recognize Tyr and its chirality in CAD. Tyr and its chirality were recognized using 18-crown-6 tetracarboxylic acid [41] and cucurbit [7] uril [42,43], where steric effects of the cages play an important role in chiral and molecular recognition. To investigate the chiral and molecular recognition of Tyr through protonation, further inspection regarding amino-acid sequences of the peptides, and structural analysis of the gas-phase noncovalent complexes are required.

**Figure 5:**
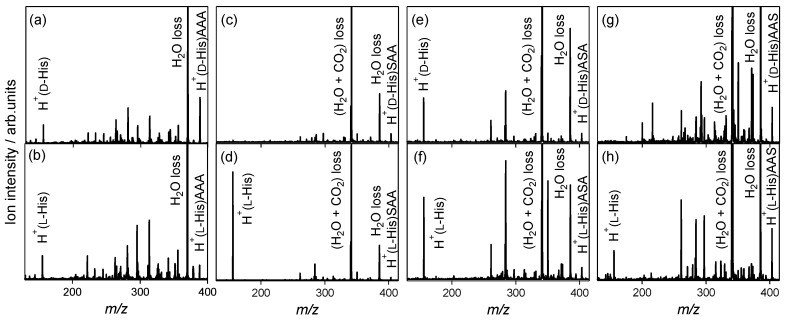
Collision-activated dissociation spectra of protonated noncovalent complexes of histidine with l-alanine and l-serine-containing tripeptides: (**a**) H^+^(d-His)AAA, (**b**) H^+^(l-His)AAA, (**c**) H^+^(d-His)SAA, (**d**) H^+^(l-His)SAA, (**e**) H^+^(d-His)ASA, (**f**) H^+^(l-His)ASA, (**g**) H^+^(d-His)AAS, and (**h**) H^+^(l-His)AAS.

His is also classified as an aromatic amino acid, which contains an imidazole side chain. The protonation site in noncovalent complexes between His and peptides could not be determined by monitoring the NH_3_ loss pathway, because no NH_3_ loss occurred in the CAD of protonated His [39]. For the l-alanine tripeptide, the H_2_O loss from the noncovalent complexes (*m*/*z* 369) and protonated His (*m*/*z* 156) are observed in the spectra of the heterochiral H^+^(d-His)AAA and homochiral H^+^(l-His)AAA (*m*/*z* 387), as shown in Figure 5a,b. Protonated His observed in the spectra indicates that proton attaches His in the noncovalent complexes. Figure 5c–h show the CAD spectra of the protonated noncovalent complexes of His enantiomers with l-serine-containing tripeptides.

In the spectra of homochiral H^+^(l-His)SAA, heterochiral H^+^(d-His)ASA, homochiral H^+^(l-His)ASA, and homochiral H^+^(l-His)AAS shown in Figure 5d–h, respectively, the H_2_O loss from the noncovalent complexes and protonated His are observed, as in the case with l-alanine tripeptide. In contrast, no protonated His is observed in the spectra of heterochiral H^+^(d-His)SAA and H^+^(d-His)AAS shown in Figure 5c,g, respectively. The enantiomer-selectivity for protonation in the noncovalent complexes is observed in the spectra for SAA and AAS. d-His does not accept protons from SAA and AAS, which indicates enantiomer-selective protonation.

#### 2.2.3. Serine Tripeptide

CAD experiments with protonated noncovalent complexes between aromatic amino acid enantiomers (Phe, Tyr, Trp, and His) and l-serine tripeptide (SSS) were also performed. The protonated l-serine tripeptide H^+^SSS is formed by the detachment of an aromatic amino acid, and no enantiomer-selectivity is observed in all the spectra (data not shown). No dissociations in the noncovalent complexes and protonated aromatic amino acids are observed in any of the spectra. This suggests that the NH_3_^+^ group of H^+^SSS is intramolecular-hydrogen-bonded with the oxygen atoms of the hydroxyl and amide groups of SSS, and the intermolecular interactions between the H^+^SSS and aromatic amino acids are smaller than those in the case of the l-alanine tripeptide and l-serine-containing tripeptides described earlier. The proton affinity of SSS is much larger than that of the aromatic amino acids in the noncovalent complexes. l-Serine tripeptide is not suitable for chiral and molecular recognition through the protonation of aromatic amino acids.

### 2.3. Chiral Recognition and Enantiomeric Excess Formation in Molecular Clouds

Gas-phase clusters are models for molecular clouds, as described in the introduction section. Photodissociation of gas-phase d-Trp via CO_2_ loss occurred when it was noncovalently complexed with l-Ser or l-Thr in the presence of Na^+^, whereas the enantiomer-selective phenomenon was not observed in the noncovalent complex with Ala [34]. The results indicate that a side-chain OH group plays an important role in chiral recognition and enantiomer-selective photodissociation, which induces enantiomeric excess in the gas phase. The chiral preference of gas-phase Ser clusters for homochirality was investigated using mass spectrometry [38]. Gas-phase Ser octamers are stable and exhibit an exceptional preference for homochirality. However, NMR and IR studies indicated that the Ser clusters do not exist in the solution [44].

Small peptides and amino acids such as Ala and Ser were formed via the UV or electron irradiation of interstellar molecules such as H_2_O, CH_3_OH, NH_3_, and HCN condensed on a cold surface [19,20,21,22,23]. The amino-acid sequences of the peptides formed in the interstellar molecular clouds are significant to the enantiomeric excess formation, because the peptides can induce the enantiomer-selective CAD of the amino acids through protonation in the gas phase. The structures determined owing to the intra- and inter-molecular hydrogen bonds are also important to understand the chiral recognition mechanism of biological molecules. To demonstrate the relations between the structures and enantiomer-selective reaction of the interstellar molecular clouds, it is useful to perform laser spectroscopy, ion-mobility mass spectrometry, and theoretical calculations for the gas-phase noncovalent complexes containing biological and interstellar molecules.

## 3. Materials and Methods 

d-Phe, l-Phe, d-Tyr, l-Tyr, d-Trp, l-Trp, d-His, and l-His (each with a purity of >98%) were obtained from Sigma-Aldrich (St. Louis, MI, USA). l-Alanine tripeptide (l-Ala-l-Ala-l-Ala, AAA) was obtained from Bachem (Bubendorf, Switzerland). l-Serine-containing tripeptides (l-Ser-l-Ala-l-Ala, SAA; l-Ala-l-Ser-l-Ala, ASA; l-Ala-l-Ala-l-Ser, AAS) and l-serine tripeptides (l-Ser-l-Ser-l-Ser, SSS) were obtained from the Toray Research Center (Tokyo, Japan). A solution containing 1 mM amino acid and 0.5 mM peptide in a mixture of water and methanol (50/50, *v*/*v*) was used. Formic acid was not added in the solutions.

The CAD spectra of mass-selected ions were obtained using a linear ion trap mass spectrometer (LTQ XL, Thermo Fisher, Waltham, MA, USA). Gas-phase noncovalent complexes of the aromatic amino acid enantiomers with protonated tripeptides were generated using nanoelectrospray ionization. The ions were transferred to the gas phase through a heated capillary and ion guides. The voltages of the ionization source, capillary, and tube lens were 2.5 kV, 11 V, and 95 V, respectively. The temperature of the capillary was 200 °C. The ions were mass-selected, dissociated, and mass-analyzed using a linear ion trap [45]. CAD experiments using a He collision gas were carried out at an activation *q*_z_ of 0.25 and an activation time of 30 ms. The collision energies were 15% and 20% (normalized collision energy provided by the instrument), where the supplemental AC voltages applied across the rods were below 1 V. The CAD spectra of 15% collision energy were illustrated in this study, because the fragment ions were identical between the collision energies.

Other instrumental parameters were a multipole 1 offset of −3.6 V, an intermultipole lens 1 of −5.1 V, a multipole 2 offset of −5.0 V, an intermultipole lens 2 of −7.9 V, a gate lens of −32.0 V, a multipole 3 offset of −7.0 V, a front lens of −5.5 V, a front section offset of −9.0 V, a center section offset of −12.0 V, a back section offset of −7.0 V, and a back lens of 0.1 V. 

## 4. Conclusions

Chiral and molecular recognition through protonation was investigated by the CAD of protonated noncovalent complexes between aromatic amino acids and l-alanine-based tripeptides using a linear ion trap mass spectrometer. The amino-acid sequences of the tripeptides required for the recognition of aromatic amino acids were determined by analyses of the CAD spectra.

AAA and ASA recognized d-Trp and l-Phe by protonation to the amino groups of the aromatic amino acids, respectively. The enantiomer-selective protonation induced an NH_3_ loss from the protonated aromatic amino acids in the noncovalent complexes, and formed an enantiomeric excess via the enantiomer-selective CAD. In contrast, d-His did not accept protons from SAA and AAS. Chirality of Tyr could not be recognized by the l-alanine-based tripeptides used in this study. SSS could not recognize the aromatic amino acids, possibly because the intramolecular hydrogen bonds of H^+^SSS were exceedingly stronger compared to the intermolecular interactions required for molecular recognition between the H^+^SSS and the aromatic amino acids.
